# Possible role of QRS duration in the right ventricle as a perioperative monitoring parameter for right ventricular function: a prospective cohort analysis in robotic mitral valve surgery

**DOI:** 10.3389/fcvm.2024.1418251

**Published:** 2024-07-04

**Authors:** Ryota Watanabe, Kotaro Hori, Keisuke Ishihara, Shogo Tsujikawa, Hideki Hino, Tadashi Matsuura, Yosuke Takahashi, Toshihiko Shibata, Takashi Mori

**Affiliations:** ^1^ Department of Anesthesiology, Osaka Metropolitan University Graduate School of Medicine, Osaka, Japan; ^2^ Department of Anesthesiology, Osaka City General Hospital, Osaka, Japan; ^3^ Department of Cardiovascular Surgery, Osaka Metropolitan University Graduate School of Medicine, Osaka, Japan

**Keywords:** cardiac surgery, mitral valve, QRS duration, electrocardiogram, right ventricle

## Abstract

**Background:**

The clinical importance of the right ventricle (RV) has recently been recognized; however, assessing its function during cardiac surgery remains challenging owing to its complex anatomy. A temporary transvenous pacing catheter is a useful tool in the small surgical field of minimally invasive cardiac surgery, and an electrocardiogram recorded through the catheter is composed of the direct electrophysiological activity of the RV. Therefore, we hypothesized that QRS duration in the RV (QRS_RV_) could be a useful monitoring parameter for perioperative RV function.

**Methods:**

We conducted a prospective cohort analysis involving adult patients undergoing robotic mitral valve repair. A bipolar pacing catheter was inserted using x-ray fluoroscopy, and the QRS_RV_ duration was assessed at four time points: preoperative baseline, during one-lung ventilation, after weaning from cardiopulmonary bypass, and before the end of surgery. At the same time points, right ventricular fractional area change (RVFAC) measured by transesophageal echocardiography and QRS duration at V5 lead of the body surface electrocardiogram (QRS_V5_) were also evaluated.

**Results:**

In the 94 patients analyzed, QRS_RV_ duration was significantly prolonged during robotic mitral valve repair (*p* = 0.0009), whereas no significant intraoperative changes in RVFAC were observed (*p* = 0.2). By contrast, QRS_V5_ duration was significantly shortened during surgery (*p* < 0.00001). Multilinear regression showed a significant correlation of QRS_RV_ duration with RVFAC (*p* = 0.00006), but not with central venous pressure (*p* = 0.9), or left ventricular ejection fraction (*p* = 0.3). When patients were divided into two groups by postoperative QRS_RV _> 100 or ≤100 ms, 25 patients (26.6%) exhibited the prolonged QRS_RV_ duration, and the mean increase in the postoperative QRS_RV_ from preoperative baseline was 12 ms (*p* = 0.001), which was only 0.6 ms in patients with QRS_RV _≤ 100 ms (*p* = 0.6). Cox regression analysis showed that prolonged postoperative QRS_RV_ duration was the only significant parameter associated with a longer ICU stay after surgery (*p* = 0.02; hazard ratio, 0.55).

**Conclusion:**

Our data suggest that QRS_RV_ duration is a useful parameter for monitoring the RV during cardiac surgery, possibly better than a commonly used echocardiographic parameter, RVFAC. An electrophysiological assessment by QRS_RV_ duration could be a practical tool for the complex anatomy of the RV, especially with limited modalities in perioperative settings.

## Introduction

The clinical importance of the right ventricle (RV) has been recently recognized in the medical management of several cardiovascular diseases and also in their surgical treatment (1–6). Emerging evidences have suggested that reduced RV function is a significant parameter for predicting higher morbidity and mortality after cardiac surgery (7–9). However, despite its importance, accurately assessing RV function is still clinically challenging because of the complex anatomy of the RV (3–5). Cardiac magnetic resonance imaging (MRI) is the gold standard for the assessment, and echocardiography is considered less accurate (9, 10), albeit currently the most practical method to evaluate cardiac function in the perioperative period.

The electrocardiographic QRS complex reflects the contraction of the heart, and its prolongation is associated with decreased cardiac function and even poor clinical outcomes (11, 12). In patients with advanced heart failure, QRS duration is a key parameter of clinical indications for cardiac resynchronization therapy (13, 14). In tetralogy of Fallot, measurement of QRS duration is recommended by clinical guidelines for risk stratification after surgical repair (15, 16), in which several reports have shown that QRS duration well correlates with RV function or volume measured by MRI (17). Electrical conduction through the right and left ventricles is known to be heterogeneous, which can be detected as small differences in QRS durations at each location of 12 leads of an electrocardiogram (ECG) (12). For example, in patients with arrhythmogenic right ventricular cardiomyopathy, which causes RV dysfunction and enlargement, the differences in QRS durations become greater with prolonged QRS durations in right-sided chest leads, such as V1–V3 (4, 18). In a small surgical field of minimally invasive cardiac surgery (MICS), a temporary transvenous catheter is a useful tool for cardiac pacing during surgery; however, when not in use for pacing, we can inversely record right ventricular ECG through the catheter. Because the ECG waveform is composed of the direct electrophysiological activity of the RV, regardless of its complex anatomy, we hypothesized that QRS duration in the RV (QRS_RV_) could be a significant parameter indicating perioperative RV function.

To address this hypothesis, we evaluated intraoperative changes in QRS_RV_ duration during robotic mitral valve repair, and examined the relationships between postoperative QRS_RV_ duration and short-term clinical outcomes. MICS causes less surgical stress (19), but it has some disadvantages for RV function, such as one-lung ventilation during surgery, difficulty in retrograde cardioplegia, or potentially inadequate de-airing after cardiopulmonary bypass. Therefore, it is possibly very important to investigate RV function in the surgical procedure.

## Methods

### Design, patients, and perioperative management

We conducted a prospective cohort study at our institution from August 11, 2019 to July 15, 2023, to evaluate the usefulness of QRS_RV_ duration as a monitoring parameter for perioperative RV function in robotic mitral valve repair surgery. The trial was approved by the Ethical Committee of Osaka Metropolitan University Graduate School of Medicine, and was registered online before patient enrollment at University hospital Medial Information Network Center (UMIN000037665). As transvenous pacing catheters were inserted in all patients who underwent robotic mitral valve surgery in our hospital, the requirement for written informed consent was waived by the ethical committee, and the study protocol was opened online to allow patients to opt-out of the study.

We enrolled 100 patients who underwent robotic mitral valvuloplasty and were aged ≥18 years at the time of surgery. The exclusion criteria were as follows: patients with Wolff-Parkinson-White syndrome, patients with implanted cardiac pacemakers, and patients in whom the insertion of transvenous pacing catheters was difficult or contraindicated (e.g., serious latex allergy). General anesthesia was induced using propofol, remifentanil, and rocuronium, and then maintained with sevoflurane, fentanyl, remifentanil, and rocuronium. During cardiopulmonary bypass (CPB), propofol was continuously administered to maintain general anesthesia. No anesthetic premedication was administered. The dose of the general anesthetic was guided by a bispectral index (BIS monitor version 4.0; Aspect Medical System Inc., Natick, MA, USA).

An arterial line was inserted into the radial artery, and the cardiac index (CI) was continuously monitored through the arterial pressure waveform using the FloTrac^TM^ system (Edwards Lifesciences LLC., Irvine, CA, USA). One-lung ventilation (OLV) was achieved using a left double-lumen tube (35 or 37 Fr Parker Endo-Bronch^TM^; Parker Medical, Inc., Danbury, CT, USA), which was initiated immediately before surgery. Perioperative drugs were administered at the discretion of the attending anesthesiologist.

### Electrocardiographic recordings

In the operating room, ECG waveforms were recorded on a server storage system throughout the procedure using a five-lead electrocardiogram (M1631A; Royal Philips, Amsterdam, Netherlands). After tracheal intubation, bipolar balloon pacing catheters (652/1–110P; Alpha Medical Instruments, Mission Viejo, CA, USA) were inserted through the right internal jugular vein, and the tip of the catheter was placed at the right ventricular apex using x-ray fluoroscopy. To record the right ventricular ECG, the bipolar tip of the catheter was connected to the limb leads of the ECG, and the difference in electrical potential between the two tip electrodes was measured using a standard limb lead system. Intraoperative QRS duration was measured at a paper speed of 50 mm/s by averaging three beats from the onset of Q wave, or the latter wave in its absence, to the end of S wave, defined as its return to the baseline (12). ECG was manually reviewed by two investigators who were blinded to the patient background. In addition to QRS_RV_, QRS duration at the precordial lead V5 (QRS_V5_) was also recorded. Twelve-lead ECG was recorded before and one week after surgery, and the QRS duration was automatically measured by averaging the durations from the 12 leads (Cardiofax G ECG-2550, Nihon Kohden, Tokyo, Japan).

### Echocardiographic examinations

A transesophageal echocardiographic (TEE) probe was inserted after tracheal intubation, and comprehensive echocardiographic examinations were performed in each patient by certified investigators of perioperative transesophageal echocardiography, who were blinded to the patient's background (Vivid E9, GE Vingmed Ultrasound AS, Horten, Norway). Right ventricular fractional area change (RVFAC) was measured as a commonly used parameter of RV function, and left ventricular ejection fraction (LVEF) was also recorded to evaluate LV function (9, 10). RVFAC was calculated as (end-diastolic – end-systolic area)/end-diastolic area × 100 (%), which was obtained by tracing the RV endocardium from the annulus to the apex in the mid-esophageal four-chamber view (ME 4CH). LVEF was measured using modified Simpson's method with ME-4CH and -2CH views. Modified TAPSE (tricuspid annular plane systolic excursion) was also measured by the difference of the distance from the RV apex to the lateral tricuspid annulus between the diastole and systole in the ME 4CH view (20, 21). This study utilized mTAPSE because obtaining original TAPSE by TEE can be difficult, owing to the different angle of the ultrasound beam in transthoracic echocardiography (TTE). Results obtained using mTAPSE reportedly correlate well with those of original TAPSE measured by TTE. Preoperative and one-week postoperative echocardiographic evaluations were performed by TTE.

### Study outcomes

The primary outcome was the intraoperative changes in QRS_RV_ duration, which were assessed at four time points: (1) baseline (Pre OP: before surgery initiation), (2) during OLV (15 min after OLV initiation), (3) after weaning from CPB (Post CPB: 15 min after protamine administration), and (4) after surgery (Post OP: during wound closure after OLV). At the same time points, QRS_V5_ and the TEE parameters, RVFAC and LVEF were also evaluated. ECG and TEE images were recorded during surgery, and the parameters were measured after patients' discharge from the hospital. Postoperative adverse events within a month after surgery were identified by a medical record review, which comprised (1) arrhythmias that were symptomatic or required treatment, (2) cerebrovascular attacks, (3) surgical revision, (4) wound infection that required surgical treatment, and (5) pulmonary complications.

### Statistical analysis

The sample size of the study was determined using G*power software (version 3.1, Heinrich Heine University Düsseldorf, Germany) to detect significant intraoperative changes in QRS_RV_ duration by an effect size of 0.2 with correlation among repeated measures of 0.3 (*α* = 0.05, *β* = 0.1). An estimated sample size of 89 patients was calculated; therefore, 100 patients were included in the study to account for potential dropouts. Continuous variables were expressed as the medians [interquartile range (IQR)] unless otherwise stated. Categorical variables were reported as numbers and percentages. Changes in QRS_RV_, RVFAC, QRS_V5_, and LVEF at the four time points were compared using repeated-measures analysis of variance on ranks with Dunn's correction for multiple comparisons. The relationships between the changes in the postoperative QRS_RV_ from preoperative baseline and those in RVFAC were assessed using a linear regression model. Further, the relationships between the QRS_RV_ and other hemodynamic parameters were assessed using a multilinear regression model. The length of ICU stay was first compared by the log-rank test, followed by the Cox proportional hazards model to adjust for the following demographic and clinical factors that were previously reported to be associated with prolonged length of ICU stay after cardiac surgery: age, sex, CPB time, and medical history of chronic obstructive pulmonary disease or atrial fibrillation (22). All statistical analyses were conducted using SigmaPlot (version 14.5, Systat Software Inc., San Jose, CA, USA). No imputation was performed for missing data, and a *p* value of <0.05 was considered statistically significant.

## Results

Of the 102 patients assessed for eligibility, 100 patients who underwent robotic mitral valve repair surgery were enrolled, 94 of whom were analyzed in the study (Supplementary Figure S1). Their clinical characteristics are shown in Table 1. The median age was 63 years, 5 patients (5.3%) were classified as New York Heart Association *III or IV*, and the median LVEF was 63% at the preoperative assessment clinic. During the surgery, the median CPB time was 188 min, and the intraoperative transfusion volume was relatively small.

**Table 1:** Patient characteristics and procedures.

	Total (*n* = 94)
Preoperative characteristics	
Age, yr, median (IQR)	63 (52–72)
Sex, male, *N* (%)	60 (63.8)
Sex, female, *N* (%)	34 (36.2)
Body mass index, kg/m^2^, median (IQR)	22 (20–24)
NYHA classification III or IV, *N (%)*	5 (5.3)
LVEF ^a^ , %, median (IQR)	63 (60–65)
Patient medical history, *N* (%)	
Af	18 (19.1)
COPD ^b^	19 (20.2)
Intraoperative care measures	
CPB time, min	188 (159–214)
Total blood loss, ml ^c^	550 (462–720)
Transfusion volume, ml	
Red cell concentrate	0 (0–0)
Fresh frozen plasma	0 (0–480)
Platelet concentrate	0 (0–0)

IQR, interquartile range; NYHA, New York Heart Association; LVEF, left ventricular ejection fraction; Af, atrial fibrillation; COPD, chronic obstructive pulmonary disease; CPB, cardiopulmonary bypass; FEV_1.0%_, % forced expiratory volume in one second.

^a^ Preoperative LVEF was measured by transthoracic echocardiography at the preoperative assessment clinic.

^b^ Medical history of COPD was decided by FEV_1.0%_ < 70% in a spirometry test or medical record review.

^c^ Total blood loss volume included the volume of intraoperative blood salvage.

### Perioperative changes in electrocardiographic and echocardiographic parameters

**Figure 1:**
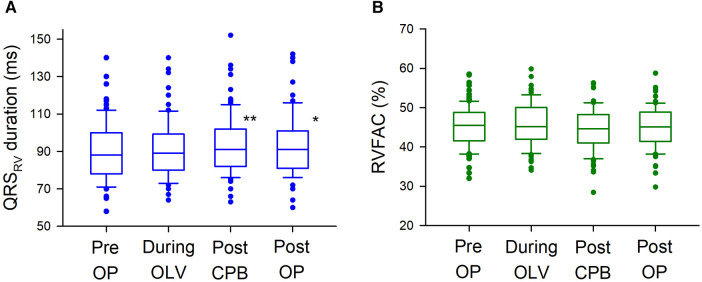
Intraoperative changes in QRS_RV_ and RVFAC. (**A**) Intraoperative changes in QRS_RV_ duration. QRS_RV_ duration was significantly prolonged after CPB weaning (Post CPB, 91 ms [82–102]; median [interquartile range], *p* = 0.0003) and after surgery [Post OP, 91 ms (81–101), *p* = 0.04] from preoperative baseline [Pre OP, 88 ms (78–100)]. The box plots show the medians (middle horizontal lines) and interquartile ranges (edges of the box). Whisker caps are set at 10% and 90% of the data, and outliers shown are the values lower or higher than the caps. (**B**) Intraoperative changes in RVFAC. There were no significant intraoperative changes in RVFAC (*p* = 0.2). QRS_RV_: QRS complex on right ventricular electrocardiogram recorded by a transvenous pacing catheter, RVFAC: right ventricular fractional area change measured by transesophageal echocardiography, ** *p* < 0.001, * *p* < 0.05.

Representative ECG recordings of QRS_RV_ and QRS_V5_ are shown in Supplementary Figure S2. Different patterns of changes in the shape of QRS_RV_ were observed in the study. Electrocardiographic and echocardiographic parameters were assessed at four time points during surgery, and the median QRS_RV_ duration was significantly prolonged during robotic mitral valve repair (*p* = 0.0009); however, there were no significant changes in RVFAC during surgery (*p* = 0.2) (Figure 1). There were also no significant intraoperative changes in mTAPSE (Supplementary Figure S3). By contrast, the median QRS_V5_ duration was significantly shortened during surgery (*p* < 0.00001), and LVEF was significantly decreased (*p* < 0.00001), albeit within normal limits on echocardiography (Supplementary Figure S4). Similar to the changes in intraoperative ECG parameters, QRS_V5_ on the 12-lead ECG at approximately one week after surgery was significantly shortened from the preoperative baseline value (*p* = 0.04), but QRS_V1_ at the right-sided chest did not change significantly after surgery (*p* = 0.06) (ECG at median postoperative day 6 [6–7] and preoperative day 2 [2–4]; median [IQR]) (Supplementary Figure S5). To examine the relationship between the postoperative changes in QRS_RV_ duration from preoperative baseline and those in RVFAC, *Δ*QRS_RV_ duration was plotted against *Δ*RVFAC (Supplementary Figure S6). There was a weak but significant correlation between *Δ*QRS_RV_ and *Δ*RVFAC (*p* = 0.004). To further examine the relationship between QRS_RV_ duration and several other hemodynamic parameters during surgery, a multilinear regression model was used, which showed a significant correlation of QRS_RV_ duration with RVFAC (*p* = 0.00006) but not with LVEF (*p* = 0.3), central venous pressure (*p* = 0.9), or CI (*p* = 0.1) (Table 2). Regarding the echocardiographic parameters on the volume of each ventricle, RVEDA (right ventricular end-diastolic area) was not significantly changed during surgery (Pre OP, 15.7 [12.8–20.3] cm^2^, During OLV, 15.9 [12.3–19.5], After CPB, 15.2 [11.4–18.7], Post OP, 14.9 [11.6–17.5]; *p* = 0.06, median [IQR]); however, LVEDV (left ventricular end-diastolic volume) was significantly decreased (Pre OP, 101.7 [92.4–11.3] ml, During OLV, 99.4 [86.7–11.8], After CPB, 59.1 [46.3–70.9], Post OP, 62.8 [52.2–79.1]; *p* < 0.00001).

**Table 2:** Multilinear regression model of QRS_RV_ duration with several hemodynamic parameters.

	Coefficient [95% CI]	*P* value
RVFAC, %	−0.76 [−1.12 to −0.41]	0.00006
LVEF, %	−0.20 [−0.60 to 0.20]	0.3
CVP, mmHg	0.02 [−1.01 to 1.05]	0.9
CI, L/min/m^2^ ^a^	2.59 [−0.72 to 5.89]	0.1

QRS_RV_, QRS duration in the right ventricle; 95% CI, 95% confidence interval; RVFAC, right ventricular fractional area change; CVP, central venous pressure; LVEF, left ventricular ejection fraction; CI, cardiac index.

^a^ CI was measured by arterial pressure waveform of the radial artery using FloTrac^TM^ system.

### Clinical correlates of prolonged QRS_RV_ duration

To investigate the association between prolonged QRS_RV_ duration and clinical outcomes after surgery, we divided the patients into two groups according to the presence of postoperative QRS_RV _> 100 ms. Perioperative data from patients with QRS_RV _> 100 ms and QRS_RV _≤ 100 ms after surgery are shown in Table 3. Twenty-five patients (26.6%) had QRS_RV _> 100 ms after surgery, with no significant differences in preoperative characteristics and intraoperative care measures. The kappa coefficient for assessing inter-rater reliability regarding group judgment was 0.81. Prolongation of QRS_RV_ duration after surgery from preoperative baseline value (*Δ*QRS_RV_) was 12 ms [6 to 19] (mean [95% confidence interval (95% CI)]) in patients with postoperative QRS_RV _> 100 ms (*p* = 0.001), whereas it was 0.6 ms [−2 to 3] in patients with postoperative QRS_RV _≤ 100 ms (*p* = 0.6). In a small number of patients, BNP (brain natriuretic peptide) was measured approximately a month after surgery; its level was not significantly different between the groups (51 [44–79] pg/ml in QRS_RV _≤ 100 ms, *n* = 21 vs. 73 [41–116] pg/ml in QRS_RV _> 100 ms, *n* = 5; *p* = 0.4).

**Table 3:** Perioperative data of QRS_RV _> 100 and QRS_RV _≤ 100 ms after surgery.

	QRS_RV _> 100 ms after surgery (*n* = 25)	QRS_RV _≤ 100 ms after surgery (*n* = 69)	Difference^a^ [95% CI]
Preoperative characteristics			
Age, yr	63 (50–71)	62 (51–72)	−0.2 [−7.2 to 6.9]
Sex, male	18 (72.9)	42 (60.9)	11.1 [−10.9 to 33.1]
Sex, female	7 (27.1)	27 (39.1)	−11.1 [−33.1 to 10.9]
Body mass index, kg/m^2^	22 (20–25)	22 (20–25)	−0.4 [−1.9 to 1.0]
NYHA classification *III or IV*	1 (4.0)	4 (5.8)	−1.8 [−12.1 to 8.5]
LVEF, % ^b^	63 (59–65)	62 (60–65)	0.2 [−2.0 to 2.5]
Patient medical history			
Af	6 (24.0)	12 (17.4)	6.6 [−11.4 to 24.6]
COPD ^c^	5 (20.0)	14 (20.3)	−2.9 [−18.7 to 18.1]
Intraoperative care measures			
CPB time, min	195 (162–225)	196 (165–220)	3 [−16 to 22]
Total blood loss, ml ^d^	581 (473–700)	594 (480–732)	24 [−122 to 170]
Transfusion volume, ml			
Red cell concentrate	0 (0–0)	0 (0–0)	−12 [−79 to 55]
Fresh frozen plasma	0 (240–720)	0 (0–480)	86 [−69 to 241]
Platelet concentrate	0 (0–0)	0 (0–0)	17 [−21 to 56]
Postoperative care measures ^e^			
P/F ratio	252 (145–340)	332 (232–408)	−52 [−116 to 11]
Intubation time in ICU, min	281 (191–999)	268 (194–943)	38 [−184 to 260]
Catecholamine index ^f^	3.3 (2.4–4.6)	3.1 (2.4–5.0)	0.07 [−1.01 to 1.15]
Laboratory data			
Peak CK-MB, ng/ml ^g^	68 (48–101)	79 (58–108)	−8 [−25 to 18]
K^+^, mEq/l	4.4 (3.8–4.6)	3.8 (3.6–4.2)	0.4 [0.2 to 0.6]
Ca^2+^, mEq/l	1.08 (1.03–1.13)	1.09 (1.04–1.13)	0.004 [−0.03 to 0.03]

QRS_RV_, QRS duration in the right ventricle; IQR, interquartile range; 95% CI, 95% confidence interval; NYHA, New York Heart Association; LVEF, left ventricular ejection fraction; Af, atrial fibrillation; COPD, chronic obstructive pulmonary disease; CPB, cardiopulmonary bypass; ICU, intensive care unit; P/F ratio, ratio of arterial oxygen partial pressure to fractional inspired oxygen; CK-MB, creatine kinase-MB; FEV_1.0%_, % forced expiratory volume in one second.

^a^ All continuous variables are expressed as the median [interquartile range (IQR)], and categorical variables are reported as the numbers and the percentages. The difference for continuous variables is shown in mean [95% CI], and that for categorial variables is shown in the absolute difference of percentage points.

^b^ Preoperative LVEF was measured by transthoracic echocardiography at the preoperative assessment clinic.

^c^ Medical history of COPD was decided by FEV_1.0%_<70% in a spirometry test or medical record review.

^d^ Total blood loss volume included the volume of intraoperative blood salvage.

^e^ Data regarding postoperative care measures was the value at ICU admission unless specified otherwise.

^f^ Catecholamine index was calculated as dopamine*1 + dobutamine*1 + adrenaline*100 + noradrenaline*100 (µg/kg/min).

^g^ CK-MB was measured immediately after surgery, at approximately 3 h, 9 h, and day 1–3 postoperatively.

As an indicator of postoperative short-term clinical outcomes, we compared the length of ICU stay after surgery between the groups, and found it to be significantly longer in patients with postoperative QRS_RV _> 100 ms (41 h [34–47] vs. 33 h [29–36], *p* = 0.02; mean [95% CI]). The period of catecholamine administration was also significantly longer in patients with prolonged QRS_RV_ duration (47 h [39–55] vs. 36 h [32–40], *p* = 0.04; mean [95% CI]). Using a Cox proportional hazards model to adjust for clinical factors previously known to affect the length of ICU stay, postoperative QRS_RV_ duration of >100 ms was the only significant parameter predicting a longer ICU stay after surgery (Table 4).

**Table 4:** Cox regression analysis for the length of ICU stay.

	Hazard ratio [95% CI]	Coefficient [95% Cl]	*P* value
QRS_RV_ > 100 ms after surgery	0.55 [0.34 to 0.90]	−0.60 [−1.09 to −0.11]	0.02
Age, yr	0.99 [0.97 to 1.00]	−0.012 [−0.028 to 0.004]	0.12
Sex, female	0.66 [0.42 to 1.06]	−0.41 [−0.87 to 0.06]	0.08
CPB time, min	1.002 [0.996 to 1.007]	0.002 [−0.004 to 0.007]	0.50
COPD ^a^	0.61 [0.34 to 1.10]	−0.49 [−1.08 to 0.10]	0.10
Af ^†^	1.36 [0.73 to 2.56]	0.31 [−0.32 to 0.94]	0.34

QRS_RV_, QRS duration in the right ventricle; CPB, cardiopulmonary bypass; COPD, chronic obstructive pulmonary disease; Af, atrial fibrillation.

^a^ Preoperative comorbidity, which was judged by physiological tests or medical record review at the preoperative assessment clinic.

### Clinical correlates of other electrocardiographic and echocardiographic parameters

Regarding RVFAC, dividing the patients by postoperative RVFAC < 35%, the absolute value of which is a frequently used cutoff value (10), resulted in only three patients (3.3%) meeting the criteria; therefore, we set the cutoff value as to 40% or 45%. For a cutoff value of 40%, 15 patients (16.7%) were included, and for a 45% cutoff, 42 patients (46.7%) were included (Supplementary Tables S1 and S2). Using both the cutoff values, the preoperative LVEF was significantly lower in patients with postoperative RVFAC of <40% or <45% than in those with RVFAC of ≥40% or ≥45%. Regarding QRS_V5_ duration, the number of patients with QRS_V5 _> 100 ms was 48 (52%), and no significant differences in preoperative characteristics and intraoperative care measures between QRS_V5 _> 100 and QRS_V5 _≤ 100 ms were observed (Supplementary Table S3).

The length of ICU stay was not significantly different between any two groups divided by RVFAC of 40% or 45% or QRS_V5_ of 100 ms (36 h [28–44] in RVFAC < 40% vs. 34 h [31–38] RVFAC ≥ 40% (*p* = 0.4); 36 h [31–40] in RVFAC < 45% vs. 34 h [29–38] in RVFAC ≥ 45% (*p* = 0.6); 35 h [31–40] in QRS_V5 _> 100 ms vs. 34 h [29–38] in QRS_V5 _≤ 100 ms (*p* = 0.6); mean [95% CI]).

### Postoperative adverse events

**Figure 2:**
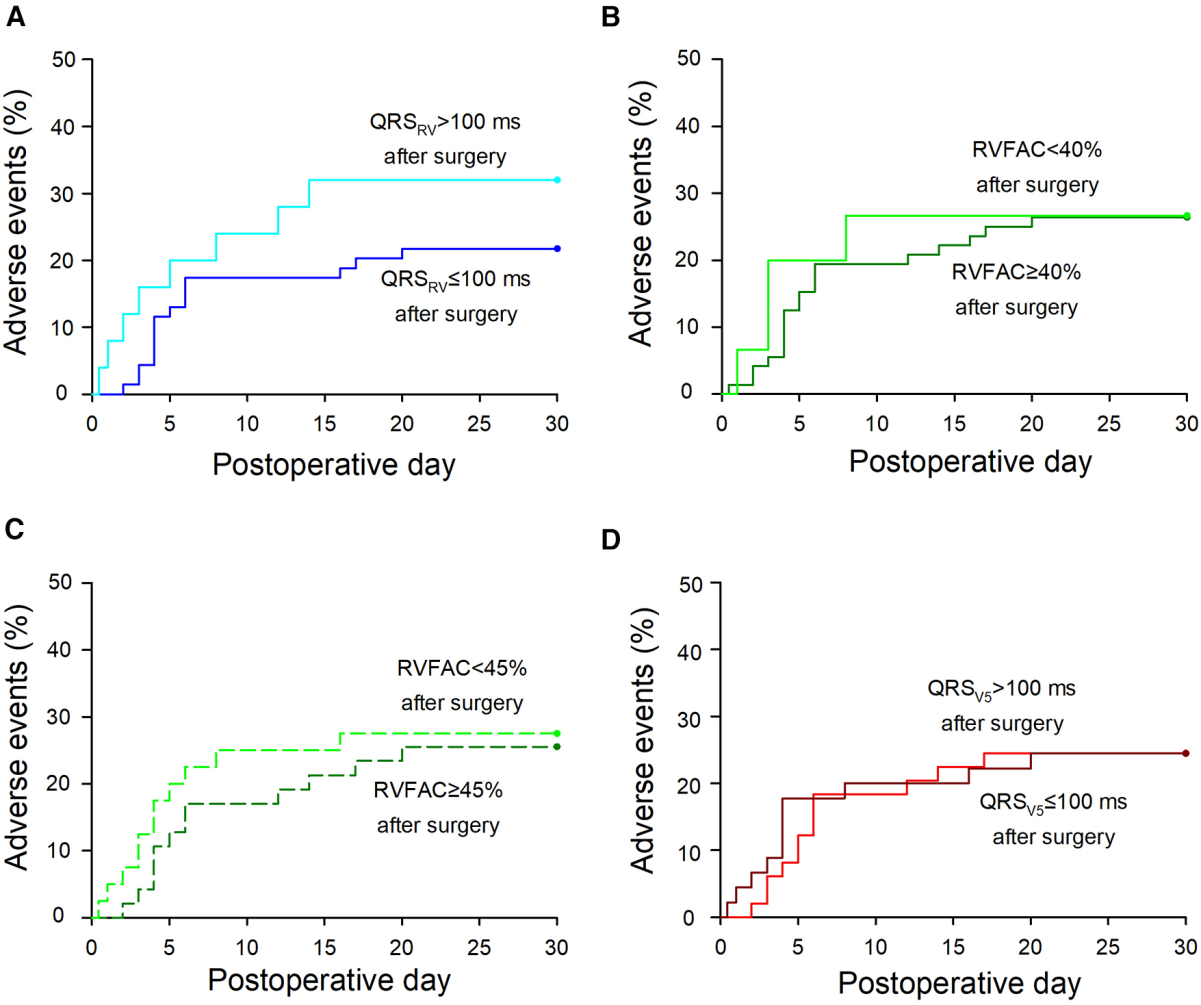
Postoperative adverse events in the groups divided by each parameter. (**A**) A Kaplan-Meier curve of adverse events in patients with QRS_RV _> 100 or QRS_RV _≤ 100 ms after surgery. The mean difference in adverse event rates between the two groups was 10.3% [−9.4 to 31.6] [mean (95% confidence interval), *p* = 0.3]. (**B**) Adverse events in patients with RVFAC < 40% or RVFAC ≥ 40% after surgery. The mean difference in adverse event rates was 0.3% [−24.8 to 24.3] (*p* = 1.0). (**C**) Adverse events in patients with RVFAC < 45% or RVFAC ≥45% after surgery. The mean difference was 2.0% [−20.6 to 16.6] (*p* = 0.8). (**D**) Adverse events in patients with QRS_V5 _> 100 or QRS_V5 _≤ 100 ms after surgery. The mean difference was 0.05% [−17.35 to 17.44] (*p* = 1.0). Details of the adverse events are shown in Supplementary Table S4. If the events occurred within 24 h after surgery, postoperative hours were converted into postoperative day (e.g., six postoperative hours were converted into 0.25 postoperative days). In patients with >1 postoperative adverse events, postoperative days of the first events were marked. QRS_RV_: QRS duration in the right ventricle, QRS_V5_: QRS duration at precordial lead V5, RVFAC: right ventricular fractional area change.

Postoperative adverse events within a month after surgery were compared between the two groups divided by each parameter. Details of the postoperative adverse events were shown in Supplementary Table S4. The mean difference in adverse event rates was 10.3% for QRS_RV_ duration (*p* = 0.3), 0.3% for RVFAC with 40% cutoff (*p* = 1.0), 2.0% for RVFAC with 45% cutoff (*p* = 0.8), and 0.05% for QRS_V5_ duration (*p* = 1.0) (Figure 2). No serious complications related to intravenous pacing catheter use were observed.

## Discussion

In this prospective cohort analysis, QRS_RV_ duration on right ventricular ECG was significantly prolonged after CPB and surgery, whereas there were no significant intraoperative changes in the echocardiographic parameter, RVFAC during robotic mitral valve repair. The mean *Δ*QRS_RV_ was 12 ms in patients with postoperative QRS_RV _> 100 ms, which was only 0.6 ms in those with QRS_RV _≤ 100 ms. Since Cox regression analysis showed that the prolonged postoperative QRS_RV_ duration was the only significant parameter for longer ICU stay after surgery, it could be clinically useful for monitoring perioperative RV function in cardiac surgery. To our knowledge, this is the first study to show the potential utility of right ventricular ECG during cardiac surgery.

Although the RV function, especially during cardiac surgery, remains unclear, several studies have shown that CPB is one of the main factors for perioperative RV impairment (23–25). Considering the high prevalence of RV dysfunction in patients with severe mitral regurgitation (up to 30%) (3, 4, 26), our data showing significant QRS_RV_ prolongation after CPB and surgery may suggest perioperatively reduced RV function during robotic mitral valve repair (Figure 1A). While multiple regression analysis or plots of *Δ*QRS_RV_ against *Δ*RVFAC showed that QRS_RV_ duration was associated with RVFAC (Table 2 and Supplementary Figure S6), intraoperative RVFAC did not show significant differences after CPB, in contrast to QRS_RV_ (Figure 1B). These findings may indicate the limitations of intraoperative echocardiography in assessing RV function. The main limitation is the complex RV anatomy, as described above, which could largely affect the measurement accuracy of RVFAC (4, 9, 10, 27). In addition, growing evidences show that the longitudinal RV contraction is often reduced after CPB even when the global RV function is preserved, suggesting that longitudinal echocardiographic measures, such as TAPSE or RV strain, are possibly not suitable for perioperative RV assessment (9, 28, 29). Global assessment of the RV is recommended; thus, we chose RVFAC as the echocardiographic parameter in this study. Three-dimensional (3D) echocardiography may improve the situation; however, especially in intraoperative TEE, measuring 3D RVEF in real time remains difficult owing to some technological issues, and does not seem to be practical at least in the current clinical settings (9, 30, 31). Since QRS_RV_ could simply indicate the electrophysiological activity of the RV, it might have bypassed the above limitations of echocardiography and might have revealed perioperatively reduced RV function after CPB, which RVFAC did not capture. Further, intraoperative changes in QRS_RV_ duration were different from those in QRS_V5_ (Figure 1 and Supplementary Figure S4), which may also indicate that QRS_RV_ could reflect the hemodynamics specifically in the RV. As described above, QRS duration is known to correlate well with cardiac function and its volume. Since cardiac function in our study appears to be reduced in both ventricles, the different trends between QRS_RV_ and QRS_V5_ duration might depend on the changes in the volume of each ventricle, as assessed by RVEDA or LVEDV with TEE. Similar trends between QRS_V1_ and QRS_V5_ were observed in 12-lead ECGs (Supplementary Figure S5), which could also support our idea that QRS_RV_ could be useful for monitoring the RV during cardiac surgery.

Similar to reports examining the prognosis of patients with QRS prolongation in left heart failure (11, 12), prolonged QRS duration in right-sided chest leads was shown to be associated with decreased RV function and poor clinical outcomes (17, 18, 32). In this study, Cox regression analysis showed that prolonged QRS_RV_ at the end of surgery was the only parameter that significantly influenced the length of ICU stay, which suggested that perioperative QRS_RV_ duration could have possible prognostic importance (Table 4). By contrast, decreased RVFAC after surgery did not significantly affect postoperative ICU stay. Interestingly, the mean *Δ*QRS_RV_ in patients with prolonged postoperative QRS_RV_ duration (>100 ms) was 12 ms, whereas it was just 0.6 ms in those with QRS_RV _≤ 100 ms. Previous studies investigated the relationships between individual increases in QRS duration and worse clinical outcomes in the general population as well as in patients with heart failure (33–36). In these reports, a QRS duration increase of only 10 ms elevated the cardiovascular risks in the study population, such as cardiac death or adverse cardiac events. Therefore, our data of *Δ*QRS_RV_ might indicate that in patients with prolonged QRS_RV_ durations, RV function was reduced during mitral valve repair, which potentially caused longer postoperative ICU stay.

This study has several limitations. First, an intravenous pacing catheter is required to assess the QRS_RV_ duration. As described above, intravenous pacing is useful in the small surgical field of MICS, with advantages in postprocedural hemostasis without the obstructive epicardial pacing wire, or requiring its placement. Pacing catheters associated with potential risks of serious complications, such as pericardial tamponade, but this risk has decreased over the years (37), and a recent analysis of more than 360,000 patients in the United States showed that the incidence rate of tamponade was 0.6% (38), which was similar to that with permanent pacemakers (39). Since the catheters were inserted using fluoroscopy in this study, the incidence could be lower, and, there were no serious complications associated with the catheters. Second, as the sample size was calculated to detect significant intraoperative changes in the QRS_RV_ duration, the calculated number of patients may not suffice to further examine the relationship between prolonged QRS_RV_ and other clinical outcomes. In Figure 2, the mean difference in postoperative adverse event rates between the two groups divided by each parameter was 10.3% for QRS_RV_ duration, 0.3% for RVFAC with a 40% cutoff, and 2.0% for RVFAC with a 45% cutoff; however, there were not significant differences in any divided groups. Although Cox regression analysis showed that prolonged QRS_RV_ duration was the only significant parameter for longer ICU stay (Table 4), a larger sample size might reveal more clinical correlates of prolonged QRS_RV_ duration. Third, because MICS requires OLV during surgery, we included measurement time points with and without OLV, which may have caused different RV overload (40). Although OLV itself did not show a significant change in the QRS_RV_ duration, it might have affected the subsequently measured data (Figure 1A).

In conclusion, this prospective cohort analysis suggests that QRS duration on right ventricular ECG could be a useful parameter for monitoring the perioperative RV in cardiac surgery. Although RVFAC is a commonly used parameter to assess RV function, our data showed that the QRS_RV_ duration is possibly a better variable for detecting intraoperatively decreased RV function and predicting short-term clinical outcomes. Considering that monitoring the RV during surgery is still clinically challenging, mainly because of its complex anatomy, a simple electrophysiological assessment of the RV based on the QRS_RV_ duration could be a practical and effective tool, especially given the limited modalities in perioperative settings.

## Data Availability

The original contributions presented in the study are included in the article/**Supplementary Material**, further inquiries can be directed to the corresponding author.
