# Supplementary material for: Possible role of QRS duration in the right ventricle as a perioperative monitoring parameter for right ventricular function: a prospective cohort analysis in robotic mitral valve surgery
Source: Front Cardiovasc Med. 2024 Jul 4;11:1418251. doi: 10.3389/fcvm.2024.1418251 (PMC11254697; doi:10.3389/fcvm.2024.1418251)
Supplement: Supplementary file 1 [file Datasheet1.pdf]

Supplementary Material

Possible role of QRS duration in the right ventricle as a perioperative monitoring parameter for right ventricular function : A prospective cohort analysis in robotic mitral valve surgery

Ryota Watanabe[1], Kotaro Hori[1*], Keisuke Ishihara[2], Shogo Tsujikawa[1], Hideki Hino[1], Tadashi Matsuura[1], Yosuke Takahashi[3], Toshihiko Shibata[3], and Takashi Mori[1]

[1]Department of Anesthesiology and [3]Cardiovascular Surgery, Osaka Metropolitan University Graduate School of Medicine, Osaka, Japan

[2]Department of Anesthesiology, Osaka City General Hospital, Osaka, Japan

*** Correspondence:**
Kotaro Hori, Department of Anesthesiology, Osaka Metropolitan University Graduate School of Medicine, 1-5-7 Asahimachi, Abeno-ku, Osaka 545-8586, Japan
Tel.: +81-6-6645-2186, Fax: +81-6-6645-2489, E-mail: k.hori@omu.ac.jp

Supplementary Table 1. Perioperative data of RVFAC<40% and RVFAC≥40% after surgery

	RVFAC<40% after surgery (n=15)	RVFAC≥40%* after surgery (n=75)	Difference** [95% CI]
**Preoperative characteristics**			
Age, yr	62 (48–69)	63 (54–72)	-4 [-12 to 5]
Sex, male	11	48	9.3 [-17.0 to 35.7]
Sex, female	4	27	-9.3 [-35.7 to 17.0]
Body mass index, kg/m^2	24 (21–26)	22 (20–24)	1.1 [-0.7 to 2.8]
NYHA classification *III or IV*	0	5	-16.7 [-49.5 to 16.2]
LVEF, %†	59 (58–63)	63 (60–65)	-3.0 [-5.6 to -0.3]
Patient medical history			
Af	3	15	0.0 [-22.2 to 22.2]
COPD‡	2	16	-8.0 [-26.2 to 12.3]
**Intraoperative care measures**			
CPB time, min	197 (153–250)	191 (165–215)	8 [-15 to 32]
Total blood loss, ml§	606 (457–631)	580 (479–727)	1.1 [-0.7 to 2.8]
Transfusion volume, ml			
Red cell concentrate	0 (0–280)	0 (0–0)	63 [4 to 123]
Fresh frozen plasma	0 (0–720)	0 (0–480)	45 [-146 to 235]
Platelet concentrate	0 (0–0)	0 (0–0)	-24 [-72 to 24]
**Postoperative care measures**§§			
P/F ratio	289 (148–397)	317 (205–399)	-22 [-99 to 55]
Intubation time in ICU, min	268 (192–1009)	256 (191–948)	1.1 [-0.7 to 2.8]
Catecholamine index¶	3.4 (2.7–5.1)	3.1 (2.4–5.0)	-0.1 [-1.4 to 1.2]
Laboratory data			
Peak CK-MB, ng/ml¶¶	72 (59–113)	70 (52–97)	0.4 [-32.8 to 33.6]
K^+, mEq/L	4.4 (3.6–4.6)	3.8 (3.6–4.2)	0.24 [-0.01 to 0.49]
Ca^{2+}, mEq/L	1.09 (1.03–1.15)	1.09 (1.03–1.12)	0.005 [-0.030 to 0.041]

*Data regarding RVFAC was missing for 4 patients (4.3%) due to the difficulty of echocardiographic assessment by the acoustic shadow of the mitral valve repair ring.

**All continuous variables are expressed as the median (interquartile range [IQR]), and categorical variables are reported as the numbers and the percentages. The difference for continuous variables is shown in mean [95% CI], and that for categorial variables is shown in the absolute difference of percentage points.

†Preoperative LVEF was measured by transthoracic echocardiography at the preoperative assessment clinic.
‡Medical history of COPD was decided by $FEV_{1.0\%}$<70% in a spirometry test or medical record review.
§Total blood loss volume included the volume of intraoperative blood salvage.
§§Data regarding postoperative care measures was the value at ICU admission unless specified otherwise.
¶Catecholamine index was calculated as dopamine*1 + dobutamine*1 + adrenaline*100 + noradrenaline*100 (μg/kg/min).
¶¶CK-MB was measured immediately after surgery, at approximately 3 h, 9 h, and day 1–3 postoperatively.
RVFAC: right ventricular fractional area change, IQR: interquartile range, 95% CI: 95% confidence interval, NYHA: New York Heart Association, LVEF: left ventricular ejection fraction, Af: atrial fibrillation, COPD: chronic obstructive pulmonary disease, $FEV_{1.0\%}$: % forced expiratory volume in one second, CPB: cardiopulmonary bypass, ICU: intensive care unit, P/F ratio: ratio of arterial oxygen partial pressure to fractional inspired oxygen, CK-MB: creatine kinase-MB

Supplementary Table 2. Perioperative data of RVFAC<45% and RVFAC≥45% after surgery

	RVFAC<45% after surgery (n=42)	RVFAC≥45%* after surgery (n=48)	Difference** [95% CI]
**Preoperative characteristics**			
Age, yr	62 (54-72)	63 (50-72)	0.8 [-5.5 to 7.1]
Sex, male	30	29	11.0 [-9.6 to 33.8]
Sex, female	12	19	-11.0 [-33.8 to 9.6]
Body mass index, kg/m^2	23 (21-25)	22 (20-25)	0.6 [-0.7 to 1.9]
NYHA classification *III or IV*	3	2	3.0 [-30.9 to 59.1]
LVEF, %†	59 (58-63)	63 (60-65)	-3.0 [-5.6 to -0.3]
Patient medical history			
Af	9	9	2.7 [-13.2 to 19.2]
COPD‡	9	9	2.7 [-13.9 to 19.2]
**Intraoperative care measures**			
CPB time, min	184 (167–238)	196 (161–212)	6 [-11 to 24]
Total blood loss, ml§	600 (483-738)	556 (395-720)	111 [-20 to 241]
Transfusion volume, ml			
Red cell concentrate	0 (0-280)	0 (0-0)	37 [-8 to 81]
Fresh frozen plasma	0 (0-720)	0 (0-480)	66 [-76 to 207]
Platelet concentrate	0 (0-0)	0 (0-0)	-11 [-47 to 25]
**Postoperative care measures**§§			
P/F ratio	297 (181–378)	321 (217–407)	-46 [-103 to 10.6]
Intubation time in ICU, min	254 (191-969)	257 (192—952)	86 [-114 to 286]
Catecholamine index¶	3.3 (2.9–5.8)	3.0 (2.2–3.9)	1.2 [0.21 to 2.1]
Laboratory data			
Peak CK-MB, ng/ml¶¶	82 (56–114)	66 (51–96)	18 [-7 to 42]
K^+, mEq/L	3.8 (3.6–4.4)	4.0 (3.6–4.3)	0.03 [-0.16 to 0.21]
Ca^{2+}, mEq/L	1.09 (1.03–1.15)	1.08 (1.02–1.11)	0.018 [-0.008 to 0.045]

*Data regarding RVFAC was missing for 4 patients (4.3%) due to the difficulty of echocardiographic assessment by the acoustic shadow of the mitral valve repair ring.

**All continuous variables are expressed as the median (interquartile range [IQR]), and categorical variables are reported as the numbers and the percentages. The difference for continuous variables is shown in mean [95% CI], and that for categorial variables is shown in the absolute difference of percentage points.

†Preoperative LVEF was measured by transthoracic echocardiography at the preoperative assessment clinic.

‡Medical history of COPD was decided by $FEV_{1.0\%}$<70% in a spirometry test or medical record review.

§Total blood loss volume included the volume of intraoperative blood salvage.

§§Data regarding postoperative care measures was the value at ICU admission, unless specified otherwise.

¶Catecholamine index was calculated as dopamine*1 + dobutamine*1 + adrenaline*100 + noradrenaline*100 (μg/kg/min).

¶¶CK-MB was measured immediately after surgery, at approximately 3 h, 9 h, and day 1–3 postoperatively.

RVFAC: right ventricular factional area change, IQR: interquartile range, 95% CI: 95% confidence interval, NYHA: New York Heart Association, LVEF: left ventricular ejection fraction, Af: atrial fibrillation, COPD: chronic obstructive pulmonary disease, $FEV_{1.0\%}$: % forced expiratory volume in one second, CPB: cardiopulmonary bypass, ICU: intensive care unit, P/F ratio: ratio of arterial oxygen partial pressure to fractional inspired oxygen, CK-MB: creatine kinase-MB

Supplementary Table 3. Perioperative data of QRS_{V5}>100 and QRS_{V5}≤100 ms after surgery

	QRS_{V5}>100 ms after surgery (n=48)	QRS_{V5}≤100 ms* after surgery (n=44)	Difference** [95% CI]
**Preoperative characteristics**			
Age, yr	62 (48–70)	64 (57–73)	-3 [-9 to 3]
Sex, male	35	23	20.6 [0.9 to 40.4]
Sex, female	13	21	-20.6 [-40.4 to -0.9]
Body mass index, kg/m^2	22 (21–25)	22 (19–24)	0.5 [-0.8 to 1.8]
NYHA classification *III or IV*	3	1	4.0 [-4.4 to 12.3]
LVEF, %†	63 (60–65)	62 (60–65)	0.5 [-1.6 to 2.5]
Patient medical history			
Af	8	10	-6.1 [-35.3 to 16.1]
COPD‡	9	9	–1.7 [-17.9 to 23.0]
**Intraoperative care measures**			
CPB time, min	196 (163–223)	197 (167–222)	-2 [-19 to 15]
Total blood loss, ml§	597 (482–739)	548 (453–715)	-43 [-175 to 88]
Transfusion volume, ml			
Red cell concentrate	0 (0–0)	0 (0–0)	-29 [-89 to 31]
Fresh frozen plasma	0 (0–480)	0 (0–720)	-81 [-220 to 59]
Platelet concentrate	0 (0–0)	0 (0-0)	20 [-15 to 55]
**Postoperative care measures**§§			
P/F ratio	307 (215–399)	328 (201–435)	-14 [-71 to 44]
Intubation time in ICU, min	256 (194–947)	908 (191–1003)	-96 [-294 to 102]
Catecholamine index¶	3.3 (2.5–4.8)	3.0 (2.3–5.1)	0.1 [-0.8 to 1.1]
Laboratory data			
Peak CK–MB, ng/ml¶¶	80 (56-106)	72 (52–105)	3 [-21 to 27]
K^+, mEq/L	3.9 (3.6–4.4)	3.9 (3.6–4.3)	0.11 [-0.08 to 0.31]
Ca^{2+}, mEq/L	1.09 (1.03–1.12)	1.09 (1.04–1.14)	-0.002 [-0.028 to 0.024]

*Data regarding QRS_{V5} was missing for 2 patients (2.1%) due to connection failure of the ECG lead.

**All continuous variables are expressed as the median (interquartile range [IQR]), and categorical variables are reported as the numbers and the percentages. The difference for continuous variables is shown in mean [95% CI], and that for categorial variables is shown in the absolute difference of percentage points.

†Preoperative LVEF was measured by transthoracic echocardiography at the preoperative assessment clinic.
‡Medical history of COPD was decided by $FEV_{1.0\%}$<70% in a spirometry test or medical record review.
§Total blood loss volume included the volume of intraoperative blood salvage.
§§Data regarding postoperative care measures was the value at ICU admission unless specified otherwise.
¶Catecholamine index was calculated as dopamine*1 + dobutamine*1 + adrenaline*100 + noradrenaline*100 (µg/kg/min).
¶¶CK-MB was measured immediately after surgery, at approximately 3 h, 9 h, and day 1–3 postoperatively.

QRS_{V5}: QRS duration of precordial lead V5, IQR: interquartile range, 95% CI: 95% confidence interval, NYHA: New York Heart Association, LVEF: left ventricular ejection fraction, Af: atrial fibrillation, COPD: chronic obstructive pulmonary disease, $FEV_{1.0\%}$: % forced expiratory volume in one second, CPB: cardiopulmonary bypass, ICU: intensive care unit, P/F ratio: ratio of arterial oxygen partial pressure to fractional inspired oxygen, CK-MB: creatine kinase-MB

Supplementary Table 4. Details of postoperative adverse events within a month after surgery

	Number of patients
Postoperative adverse events	23
Arrhythmias	17
Af*	9
SVT†	2
VT	1
CAVB, SSS	5
CVA	1
Surgical revision of hemorrhage	1
Wound infection‡	2
Pulmonary embolism	1
Pneumonia	1

* Af was defined as persistent or frequently recurrent for >48 hours in patients without preoperative Af

† SVT was defined as regular tachycardia with narrow QRS complex that was symptomatic or required treatment.

‡ Wound infection was defined as a condition that required surgical treatment.

Af: atrial fibrillation, SVT: supraventricular tachycardia, VT: ventricular tachycardia, CAVB: complete atrioventricular block, SSS: sick sinus syndrome, CVA: cerebrovascular attack

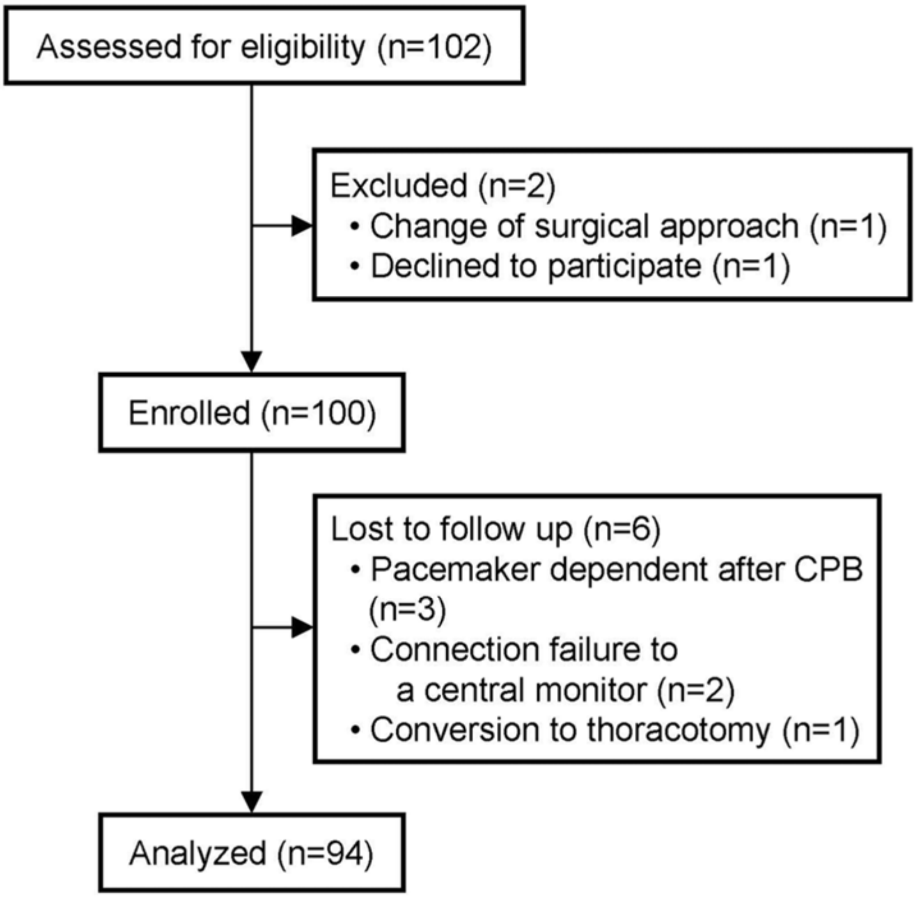

Supplementary Figure 1. Enrollment and analysis of patients in the study

A.

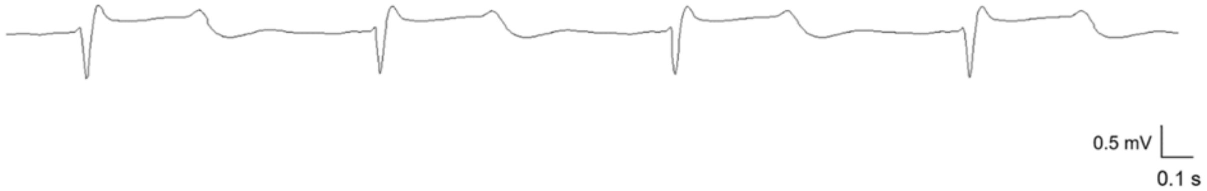

B.

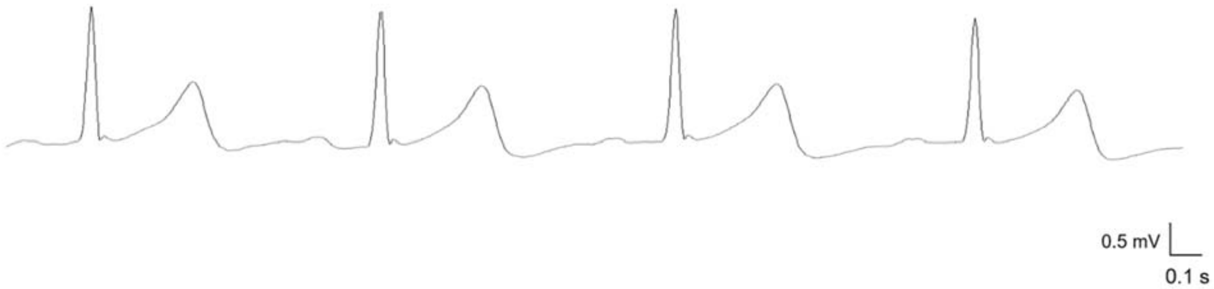

Supplementary Figure 2. Representative electrocardiograms in the right ventricle and at the precordial lead V5.

A. Representative electrocardiogram (ECG) in the right ventricle (RV). There were different patterns of changes in the shape of QRS in the RV. **B.** Representative ECG at the precordial lead V5. ECG at V5 lead was recorded at the same time points as the ECG recordings in the RV.

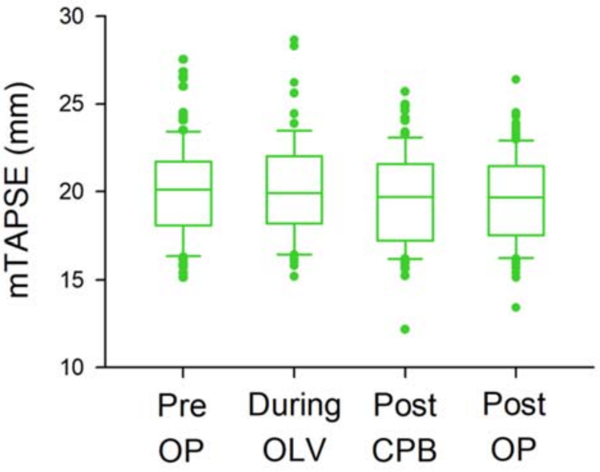

Supplementary Figure 3. Intraoperative changes in modified TAPSE

There were no significant intraoperative changes in mTAPSE (Pre OP, 20.1 [18.1–21.7], During OLV, 19.9 [18.2–22.0], Post CPB, 19.7 [17.2–21.6], Post OP, 19.7 [17.5–21.5]; p=0.07). mTAPSE: modified tricuspid annular plane systolic excursion measured by transesophageal echocardiography in the mid-esophageal four-chamber view.

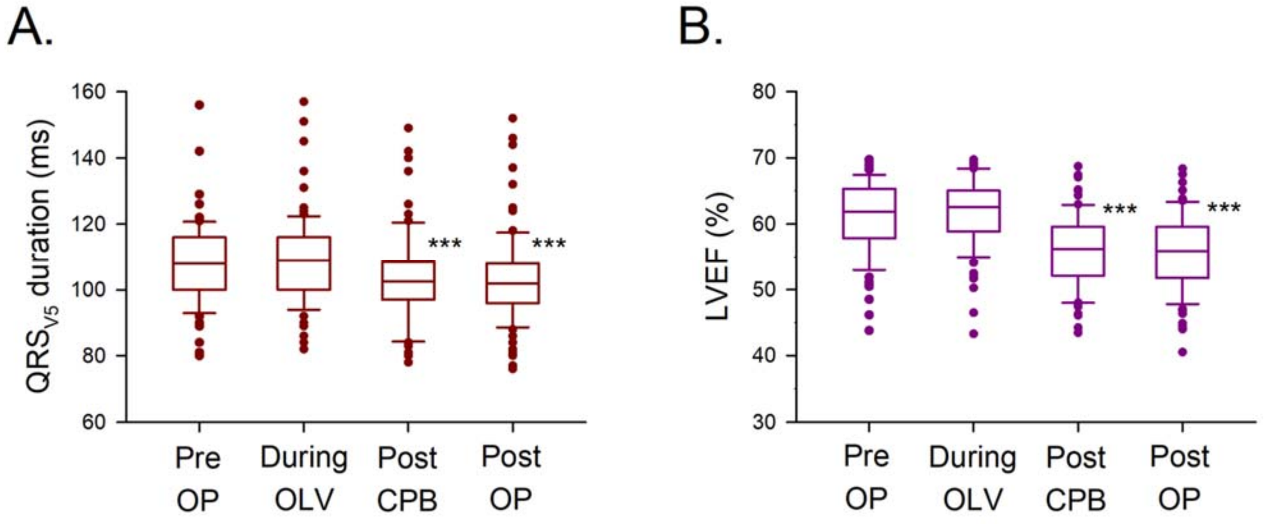

Supplementary Figure 4. Intraoperative changes in QRS_{V5} and LVEF

A. Intraoperative changes in QRS_{V5} duration. QRS_{V5} duration was significantly decreased after CPB weaning (Post CPB, 103 ms [97–109]; median [interquartile range], p<0.00001) and after surgery (Post OP, 102 ms [96–108], p<0.00001) from preoperative baseline (Pre OP, 108 ms [100–116]). **B.** Intraoperative changes in LVEF. LVEF was significantly decreased after CPB weaning (Post CPB, 56% [52–60], p<0.00001) and after surgery (Post OP, 56% [52–60], p<0.00001) from baseline (Pre OP, 62% [58–65]). QRS_{V5}: QRS duration at precordial lead V5, LVEF: left ventricular ejection fraction measured by transesophageal echocardiography, *** p<0.0001.

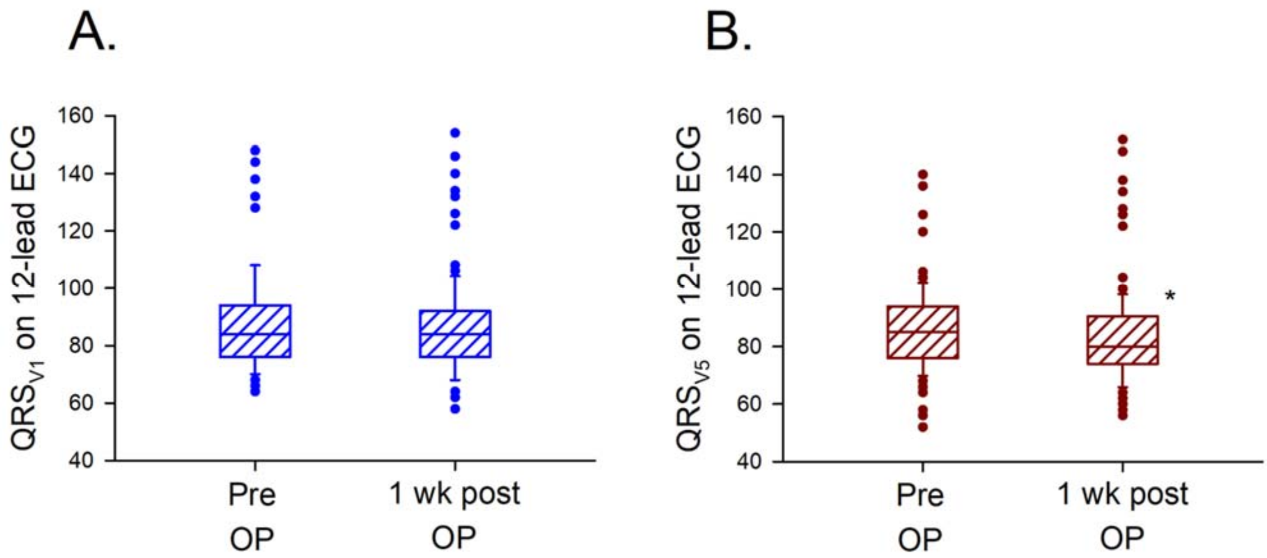

Supplementary Figure 5. Perioperative changes in QRS duration at V1 and V5 leads of 12-lead electrocardiogram

A. QRS_{V1} duration on 12-lead electrocardiogram before surgery and at one week after surgery. QRS_{V1} duration was not significantly changed at one week after surgery from preoperative baseline ($p=0.06$). **B.** QRS_{V5} duration on 12-lead electrocardiogram before surgery and at one week after surgery. QRS_{V5} at one week after surgery was significantly shortened from baseline ($p=0.04$). QRS_{V1}: QRS duration at precordial lead V1, QRS_{V5}: QRS duration at precordial lead V5, ECG: electrocardiogram, * $p<0.05$.

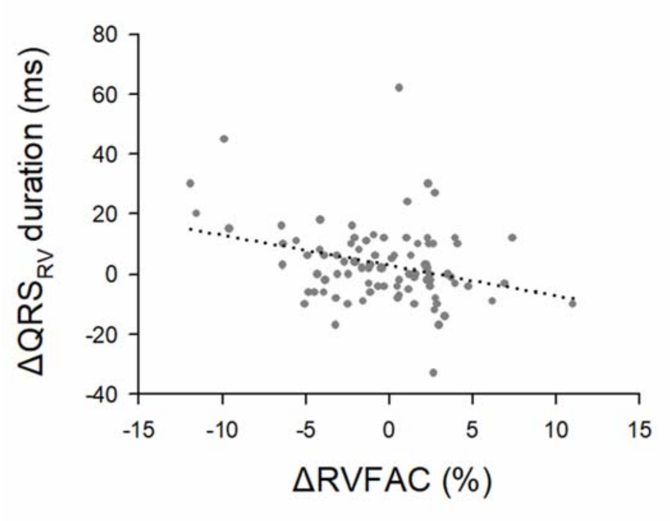

Supplementary Figure 6. The relationships between ΔQRS$_{RV}$ duration and ΔRVFAC

ΔQRS$_{RV}$ duration (changes in postoperative QRS$_{RV}$ duration from preoperative baseline; y-axis) is plotted against ΔRVFAC (x-axis). The formula of the regression line is $Y = 2.95 + (-0.99 \times X)$ (dotted line), and the correlation coefficient (R) was 0.3 ($p = 0.004$). QRS$_{RV}$: QRS complex on right ventricular electrocardiogram recorded by a transvenous pacing catheter, RVFAC: right ventricular fractional area change measured by transesophageal echocardiography.
